# Under consent: participation of people with HIV in an Ebola vaccine trial in Canada

**DOI:** 10.1186/s12910-021-00606-6

**Published:** 2021-04-09

**Authors:** Pierre-Marie David, Benjamin Mathiot, Oumy Thiongane, Janice E. Graham

**Affiliations:** 1 Faculté de Pharmacie, Université de Montréal, C.P. 6128, Succursale Centre-Ville, Montreal, H3C 3J7 Canada; 2 Département d’anthropologie, Université de Montréal, Pavillon Lionel-Groulx C. P. 6128, Succursale Centre-Ville, Montreal, QC H3C 3J7 Canada; 3 Pediatrics (Infectious Diseases), Faculty of Medicine, Dalhousie University, Halifax, B3H 4H7 Canada

## Abstract

**Background:**

Little is known about volunteers from Northern research settings who participate in vaccine trials of highly infectious diseases with no approved treatments. This article explores the motivations of HIV immunocompromised study participants in Canada who volunteered in a Phase II clinical trial that evaluated the safety and immunogenicity of an Ebola vaccine candidate.

**Methods:**

Observation at the clinical study site and semi-structured interviews employing situational and discursive analysis were conducted with clinical trial participants and staff over one year. Interviews were recorded, transcribed and analysed using critical qualitative interpretivist thematic analytical techniques. Patterns were identified, clustered and sorted to generate distinct and comprehensive themes. We then reassembled events and contexts from the study participants’ stories to develop two ideal portraits based on "composite characters" based on study participants features. These provide ethnographically rich details of participants’ meaningful social worlds while protecting individual identities.

**Results:**

Ten of the 14 clinical trial participants, and 3 study staff were interviewed. Participant demographics and socio-economic profiles expressed limited contextual diversity. Half were men who have sex with men, half were former injection drug users experiencing homelessness, one was female, none were racialized minorities and there were no people from HIV endemic countries. Fully 90% had previous involvement in other clinical studies. Their stories point to particular socio-economic situations that motivated their participation as clinical labor through trial participation.

**Conclusions:**

Our findings support Fisher’s argument of “structural coercion” in clinical trial recruitment of vulnerable individuals experiencing precarious living conditions. Clinical trials should provide more detail of the structural socio-economic conditions and healthcare needs which lie “under consent” of study participants. Going well beyond an overly convenient narrative of altruism, ethical deliberation frameworks need to sufficiently address the structural conditions of clinical trials. We offer concrete possibilities for this and acknowledge that further research and clinical data should be made available underlying study participant contexts with regards to recruitment and participation in resource poor settings, in both the South and the North.

**Supplementary Information:**

The online version contains supplementary material available at 10.1186/s12910-021-00606-6.

## Background

The concept of “ethical variability” troubles the way ethical informed consent often neglects socioeconomic and other contexts that can shape study participant’s conception, engagement and interaction in clinical trial research [1]. Ethical variability is mobilised to acknowledge and contrast the various ways in which clinical research is conducted in Northern and Southern settings. This distinction is not always operationally effective though, as conditions attributed to the "global south" can also be found in the north; vulnerable or disadvantaged people are everywhere and equally experience clinical research as a form of "professional" labor to access necessary resources [2]. Informed consent is considered in principle as a relational action to inform and make potential study participants aware of the intention and procedures of the experimental research. It is intended to provide individuals with knowledge about the potential harms of their trial participation so they understand the balance of harms and benefits. From another perspective, consent can be seen as a procedural act compliant with research ethics requirements. As such, consent appears as a type of contract which enables stakeholders to do what is expected in a clinical trial while authorising a form of clinical labor [3]. Exceptional times, during pandemics (COVID-19) or pandemic threats (Ebola) for instance, can lead to amendments to study standard operating procedures, especially concerning consent [4]. Little is known about what lies "under consent" in such a context, especially when it involves research in diverse North and South settings.

In August 2014, during the West African Ebola outbreak, the World Health Organization declared a Public Health Emergency of International Concern (PHEIC), catalysing the international community to rapidly fund development of Ebola treatments and vaccines [5]. The epidemic spurred accelerated clinical research as epidemic response [6] in particular in African settings [7], prompting the global health community to develop new vaccines and financial mechanisms to address the current and future infectious diseases. The capacity of vaccines to prevent infectious diseases was fundamental to international initiatives, but so too was assuaging the public health burden of care and management of such diseases in low income countries not to be missed [8].

In an effort to gain enough data to license a vaccine, Ebola clinical trials have continued to be conducted in sites around the world, both during and after the West African outbreak. The methodologies and ethics of these trials demand “thoughtful engagement" with research emerging from extraordinary circumstances, especially the challenges surrounding structural inequalities in North–South partnerships [9, 10]. Debates, for example, have raised procedural concerns [11]. European-African collaborations "have been prolific" [12], yet they generally involve northern technologies and institutions, and southern research subjects. The WHO-Guinea Ebola clinical trial, for instance, involving research subjects in the south during the crisis [13], serves as a touchstone of many of these issues [5]. Whereas participant recruitment in non epidemic contexts in Africa has been studied [14], little is known about enrolment of volunteers in northern settings for vaccine clinical trials of highly infectious diseases with no approved treatments.

In 2014, shortly after the PHEIC declaration, Merck, Sharpe & Dohme Corp (hereafter “Merck”) bought the patent rights for the promising *r*VSV ZEBOV vaccine and proceeded to establish a series of private–public partnerships to conduct the necessary clinical trials to gain licensure for the vaccine. Originally developed by the Public Health Agency of Canada, *r*VSV-ZEBOV is a recombinant attenuated form of the vesicular stomatitis virus—a rhabdovirus—expressing the glycoprotein of the Ebola virus. Phase I clinical safety trials of the Ebola *r*VSV ZEBOV vaccine candidate were conducted in Europe and Africa [15] as well as in North America [16, 17], funded by national agencies, e.g. the Public Health Agency of Canada (PHAC), and the Canadian Institutes of Health (CIHR) as well as the US Department of Defense Joint Program Executive Office for Chemical and Biological Defense Medical Countermeasure Systems’ Joint Vaccine Acquisition Program (MCS-JVAP) under contracts to the US Army Medical Research Institute of Infectious Disease (USAMRIID) and Battelle Biomedical Research Center.

## The altruism hypothesis and the aim of the study

One of these Phase I clinical trials was conducted in Canada in 2014. In the heat of Ebola media attention, there was a "rush to participate" with an overwhelming number of requests from the local community. More than 300 people expressed interest in volunteering the first days after the study was made public and without advertisement, although only 40 were needed [18]. Among other motivations, "altruism" was reported to explain this unprecedented volunteer response motivated by a sense of “community responsibility” and “identification with the community affected” [18].

In 2016, Merck, the International Development Research Centre (IDRC), and the Canadian Immunization Research Network (CIRN), obtained a grant from PHAC and CIHR. The Phase II Canadian African Trial for Ebola Vaccine (CATEbola)^1^ study was to be conducted in adults with HIV at four sites in Africa and Canada in order to evaluate the safety and immunogenicity of an Ebola vaccine candidate in HIV-infected adults and adolescents.

In this article, we ethnographically explore the motivations of Canadian HIV immunocompromised participants to engage in a Phase II Ebola clinical trial. Were they different from the Phase I trial also conducted in Canada? Who were the participants, what were their lives like and how did they account for their participation in the trial? What lies "under consent" according to their personal histories, discursive narratives, understanding of, and engagement with the trial. Additionally, since motivations are shaped by social relations [19, 20], we considered the perspectives of the clinical research staff, its investigators, research personnel, and assistants. We were interested in the "trial community" [19], in the social and moral relationships configured among the trial of participants, researchers, including other considerations they might hold (i.e., friends infected with HIV, Africans who were geographically at greater risk of Ebola, and non-human objects such as the exchange of money, blood and drugs involved in the scientific study).

## Methods

### The study

Our qualitative study of participants’ motivations to “volunteer” for an Ebola vaccine trial in a northern setting is part of a larger socio-anthropological project on vaccine development. *Global Vaccine Logics* was a multinational CIHR-funded project.^2^ The GVL study aimed to generate and analyze accounts of Ebola vaccine research interventions among community members, health care providers (formal and informal), biomedical researchers, government officials, NGOs and multilateral organizations. This article provides an anthropological account of one Canadian site of the CATEbola study.

### The setting and its dynamics in the clinical trial

The clinical study took place in an AIDS Research and Care Hospital Unit in Canada, a clinical care center for HIV positive people and concurrently, a clinical research center which recruits subjects. The clinical investigators were initially enthusiastic; the site had considerable experience with HIV clinical trials and the highly successful recruitment experienced during the 2014 Phase I Ebola vaccine trial gave the study coordinators reason to believe that the Phase II recruitment would be similar.

Initially, the CATEbola study investigators intended to recruit HIV positive people with (1) CD4 above 500 /ml, and (2) CD4 > 350/ml and < 500/ml from the Canadian sites. The following 3 phases involving individuals with lower CD4 cells and a dose variation were to take place at the two African sites.

The study protocol involved 8 appointments; one for eligibility, a second for the placebo or vaccine administration, and 6 follow-up visits over the 12 months following injection. Reimbursed for expenses during each visit, full completion of all 8 trial visits provided a total compensation of CAN $440 for the trial participants.

### Methods, data collection and analysis

We recruited participants through the clinical study—their research nurse presented our ethnographic study to trial participants, mentioning a $25 “compensation” per interview. If the prospective participant accepted, an interview was scheduled. We explained during the interviews that we were not part of the clinical team and that this was a separate study to investigate their motivations for participation in the trial.

Semi-structured interviews were conducted (interview guide in Additional file 1), using a situational and discursive analysis methodological approach [21, 22]. Two clinical research nurses as well as a clinical investigator were also interviewed. Interviews took place from December 2018 to November 2019 and were recorded, transcribed and translated if necessary. Analysis was conducted manually by the team in both French and English. Interviews were scrutinized for patterns that were identified, clustered and sorted until distinct and comprehensive themes were generated using an interpretivist approach [23]. Data were first discussed and analysed by the two first authors and then with the research team during regular teleconference meetings and at a workshop in Halifax in June 2019.

## Results

### Unexpected recruitment difficulties

We interviewed 10 of the 14 trial participants. The trial encountered major delays due to recruitment issues. Despite the research study’s location in a clinical care centre, we observed minimal clinical *care*. Instead, clinical trial *work* revolved around administration of a lengthy recruitment process. Over 1500 patient medical files were screened to arrive at 14 eligible participants. For the experienced trial team, this slow recruitment posed particular and unexpected problems.

The CATEbola clinical team had expected to recruit, as they ordinarily did, from patients receiving their medical care at the Centre. Instead, they had to reach beyond those patients to "the community". They adopted a new recruitment strategy, placing advertisements in other care units and remarkable to them, in the gay press and community-based organizations that provided services, for example, to people experiencing homelessness.

This revised recruitment strategy had consequences for the trial and resulted in a high proportion of former injection drug users (IDUs) in the study population. The eligibility criteria regarding illicit drug use needed careful monitoring. According to protocol, eligible participants were not to have used any street drugs in the previous six months and during the trial. This criteria proved challenging to follow, as did some participants. Several phone calls were often necessary, including to community organizations, to ensure attendance at appointments for participants with precarious residential arrangements or without a phone. These follow-ups added to the clinical labor of keeping the participants engaged in the trial.

### Participants' context and background

Four of the 14 potential interviewees could not be reached by the nurse or were unwilling to participate. Participant demographics and socio-economic profiles show limited diversity. They were older than the general population and 9 of the 10 were men. While half the study population were men who have sex with men (MSM), the other half were injection drug users (IDU) experiencing homelessness. These characteristics are not generalizable to the wider population of people living with HIV in this Canadian city. All participants were born in Canada, none were from HIV endemic or Ebola-affected countries.

Four of the ten participants were recruited directly from the HIV care center that delivers services and provides medications and regular follow-up where the trial site was located.

### Participant composite accounts

The precarious living conditions and social deprivation experienced by a significant portion of people interviewed was striking, providing contextual depth underneath their motivation to participate in the trial. This was not the first time most of these volunteers had been clinical trial subjects. Almost all—9 out of 10—had already participated in other, mostly HIV, clinical studies.

Adopting Bluebond-Langner’s (1980) methodological approach to build "composite characters", that is to say, gathering and then assembling typical or revealing characteristics that profile and portray the HIV positive people in the study [24], we aimed to preserve confidentiality by de-identifying individuals in the telling of their stories. We amalgamated accounts and compiled two sample portraits that represent characteristics and personalities of the variety of people we interviewed to explore and analyse their motivations. Eric^3^ and Camilio are composite characterizations of the 10 trial participants.

Eric is an English-speaking gay man. Diagnosed with HIV in the late ‘80 s, Eric deplored the idea of dying while still working for a boss, and so he lived on a farm with his boyfriend for 20 years until having to move to the city for health reasons. This was his first involvement in a clinical trial, having been unable to participate previously due to the geographical distance. He described his motivations:

“Because you know you sort of... Ebola is nothing you want to play around with too much. And it concerned me a bit. But I figured they know what they are doing. (…) . So you know I felt, you know if I could help out anyway, I'd like to. And here, there is lots of studies. So you know I can have a look at what's available, and whatever, and choose to do a few that are attractive. And also, the monetary aspects of it are quite appealing.”

“Well I just... People conducting clinical trials should, have figured it out that they are not going to damage us. It involves a lot of faith (laugh) in their... in their skills and that, so.”

“She [study nurse] answered the questions fine and I signed it so… That was all dealt with.”

“If they had not given me any money, I wouldn't have done the study.”

Despite mentioning his altruistic desire to participate in clinical trials, Eric emphasized that the money was his primary incentive. The consent and his signature legitimized the contract and obligations to the trial.

Camilio, a Canadian in his 40 s, said he was a former injection drug user. He had experiences as a sex worker, drug dealer, and with homelessness and imprisonment, and was diagnosed with HIV in prison in 1999. He had been regularly following HIV treatment for three or four years and participated in this clinical trial despite his physician’s disapproval, seeing it as an opportunity:

“The walls are lined with that! You know places for people to get money. You know?”

“Well, to say from the beginning, there was money. It was fucking important”

“Well, I thought I like that. If I can participate in research for drugs like Ebola. If I can participate in making a change for the medication. You know, I always wanted … I've always given … blood vials here and there, research. If you need it, I can, I'll do it. If it can help us [HIV+ people] heal, I would have done my part. You know because it's not obvious there. In the ‘80s they would die of HIV [he snaps his fingers]. You know today we are good with the drugs we have. Then its getting better and better. Then I want to be part of it. As long as you live with it, can you make a difference? As long as I live with it, I can't just keep it for myself … I'll do something with it? I do not just want to live with it and then … No. It's a way of … acceptance too. At least I would have made a difference, you know. I made a difference in the streets for 20 years (laughs)! And now I'll make a difference, but a better one. No it's my part, it's doing my part.”

Camilio needed the trial money and has participated in several studies. The clinical trial emerges here as a tool for redemption; by reversing his motivations from “money-related” to “helping others-related”, he echoes other forms of "confessional technologies" linked to biomedical access [25]. Both are very important motivations for Camilio, who unlike others in the study, participated despite his doctor’s concern given his very low CD4 count. He said that taking this risk to his health strengthened his moral commitment to the trial. This position differed from that offered by most people interviewed, who enjoyed what the research team called "VIP care", with easier access to nurse and doctors, closer follow-up and better access to care.

### Clinical labor and altruism

There were two dimensions pertaining to ethical variability that mark our findings compared to the 2014 Phase I Ebola trial: (1) temporality and perceived urgency—the 2014 Phase I trial took place during the Ebola epidemic when there was no known vaccine creating an urgency that was past tense by 2018–2019 and this Phase II trial of immunocompromised HIV participants; and (2) participants’ socioeconomic contexts—the altruistic response reported in the 2014 trial emphasized "community responsibility" [18], with Canadians volunteering to participate in a vaccine trial against a disease that did not directly threaten them. This global community responsibility was very different from that described by our trial participants four years after the West African Ebola outbreak had ended. Most participants referred to their past experiences in clinical trials when discussing their motivations. If altruism was a major motivation to participate during the international media spotlight of Ebola in 2014 [18], our research hints at precarity in understanding the reason for participating of CATEbola study volunteers, linked to their HIV status, the way they experience treatment, and their fragile socio-economical backgrounds.

The participants described two types of engagement in a trial that provided them with extra material resources. First, gay participants voiced therapeutic activism, representing themselves as members of a community that has benefited from experimental treatments along a collective therapeutic journey and care path; they wanted to do their part in advancing scientific treatments for their own community, or for people exposed to other infectious disease risks, such as Ebola. All *said* they would have participated if there had been no financial incentive. On the other hand, participation of former IDUs took the form of personal redemption; it provided an opportunity to make a positive difference for some. Still, they all needed the extra money that the trial provided. We found two different moral and social engagements in the CATEbola trial related to the social history of HIV infection in Canada; two social and moral worlds which seldom crossed during the trial. Although one participant navigated both social worlds, the trial itself did not contribute to any kind of social ideal of a trial “community”. Clinical labor was by far the most common experience of trial participants rather than that of a community of practice.

Nine out of the 10 participants had volunteered in previous clinical trials. Indeed, the trial participants frequently discussed the value of their labor, the way they reorganized their time for the trial, how they behaved in a manner that provided effective data and offered their body parts, for example, blood samples. The research nurse worked closely with IDUs to complete the questionnaire about their symptoms following injection of the experimental vaccine; the detailing of their bodily sensitivities were positively encouraged as it was valued in the production of trial data to register every possible effect of the injection or the placebo. One of the clinical trial team member told us that former IDUs had good skills in understanding their body sensitivities, especially concerning the injection’s side effects. Most of the gay participants, who had many years of antiretroviral treatment behind them, were viewed as “good” participants by study personnel, well experienced in making the connections between their embodied feelings, signs, and symptoms to the bureaucratic clinical trial questions. This linking of the body to the bureaucratic regimen of the trial to account for the effects of the trial intervention were a form of "embodied labor" [3]. Indeed, the recognition of clinical labor was a common experience repeated in interviews with participants as well as the staff. When questioning this labor, the consent form appeared as an umbrella that justified this clinical labor relationship, comparable to a work contract. In fact, consent was experienced by most of the participants more like a contract than a deliberative process of informed consent.

## Discussion

### An African–Canadian relationship?

There was little evidence from the participants interviewed of any notion of African–Canadian relationships that was prominent in the description of the trial by the study investigators. Our results show very little knowledge or interest from the participants about Ebola epidemics in Africa, nor was any mention of being part of a common experience reinforced by the clinical trialists. The international partnership involving Canadian and African sites was about a research opportunity, rather than identifying or constructing any common, one world experience among study participants. This was the first international trial lead by the Canadian team; an opportunity to put their Canadian institution on the map of global vaccine studies in the UK, the US, Germany, Belgium and France. In contrast, the research centres in Africa have a rich history of clinical research during colonial times and post independence. Further research is forthcoming that assesses the motivations of Africans participating in the trial, how they interpreted their connection to this Afro-Canadian research and how this research was experienced and meaning-making for them.

### Beyond the convenient narrative of altruism, how to address the structural conditions of clinical trials

Major delays in the trial were experienced because of unforeseen recruitment problems in Canada that had international consequences. Awaiting approval to study the safety and immunogenicity of the *r*VSV-ZEBOV vaccine in vulnerable adults and adolescents with HIV, delayed the study start. In the end, it took two years for the Canadian research site to recruit 14 of the 25 participants originally expected. The contrast between the 2014 Phase I trial response during the Ebola outbreak and this Phase II trial in 2018 signals the importance of considering temporality and perceived urgency. Outside of Africa, the Ebola outbreak in the Democratic Republic of Congo starting in the summer of 2018 did not spark the same interest as the West African epidemic, perhaps due to the decline in a pandemic threat. Forthcoming comparisons regarding the two other research sites in Africa will clarify clinical and ethical variability in the trial. Delays in completing the CATEbola study resulted in a missed opportunity to provide study results by December 2019, when the Merck Ebola vaccine [26] was licensed by the US FDA, and by 4 African countries in February 2020 [27]. Various explanations can account for this delay, the most disappointing perhaps being the failure of altruistic motivation to participate that had been reported from the earlier trial. Voluntariness to participate in a clinical trial should not be taken for granted whether in the South or in the North. More fundamentally, these results have important implication for bioethics, as described by Jill Fisher: “Rather than worrying so much about the possibility of research to exert undue influence over participants, the field of bioethics must examine the ways in which the research enterprise is embedded in broader political, economic, and social contexts that pattern who is likely to view study participation as valuable” [28].

More careful understanding is needed of the reasons for and contexts in which people decide to participate in a clinical trial. This is especially important for those experiencing socio-economic vulnerability. Jill Fisher describes "structural coercion" which “shifts the frame of ethical deliberation away from specific individuals and specific studies to see important patterns in research participation by salient demographic characteristics” [28]. An alternative worth consideration would co-produce both study recruitment and consent, including the communities targeted by the research in negotiating the research process from its earliest stages and through the various study visits. In this way, study participants, individually and collectively, could have a greater role and investment in the process, with the capacity to negotiate and reaffirm the terms of consent at various moments in the trial and question the terms of their clinical labor. Discussions surrounding the renewal of consent at every visit could be an avenue, particularly relevant in a context where participants’ clinical work is akin to "drudge" work. More fundamentally, however, participants should be allowed to build a balance of power, defend their conditions and claim a sort of emerging labor law if their "clinical work" is to be taken seriously. As a result, participants should be allowed a platform to share their experience in order to foster collective representation regarding their clinical labor conditions. Clinical trials should offer conditions besides confidentiality and respect for autonomy, which often lead to unquestioned individualization of participants, preventing them from a shared experience with potential for greater collective action in the face of structural coercion. This would be a much more deliberatively democratic consensual process than the "once for all" signing of an informed consent contract.

## Limitations

Our team’s lack of control over the recruitment process, which was managed by the clinical study personnel as a condition of our access, was a limitation to this ethnographic study. We relied on the clinical team for access to trial participants. By the time we could interview, most had already completed the trial. We were unable to interview or access information about motivations of those refusing to participate in the study.

## Conclusion

We examined what lies under consent as a research object to better understand motivations to participate in an Ebola clinical trial for individuals with HIV. The diverse intersectional range of social, economic and temporal contexts of clinical trial participants must not be taken for granted in biomedical research. We found that the social contexts in which Canadian participants live, the way they experience HIV infection, as well as their medical treatments and research engagements, affected their motivation to participate in a Ebola vaccine trial in 2018. We suggest that "motivations", as understood as rational individual choices, can too easily be reduced to explanatory models such as "altruism". Such explanations may hide the multiplicity of contextual complexities that motivate people to volunteer for clinical trials. Presenting the motives of Canadians who participated in an Ebola clinical trial in 2014 as "altruistic" may overshadow the contexts and actual realities motivating participation in both highly developed and less developed countries. Further research is necessary to address these issues and throughout clinical trial research in resource poor settings, whether in the North or South, where structural inequities affect access to care and enable forms of coercion that are not addressed under current framings of ethical deliberation.

## Supplementary Information

**Additional file 1.** Interview guides.

## Data Availability

The datasets generated and analysed during the current study are not publicly available due to further comparative investigations but are available from the corresponding author on reasonable request.

## Footnotes

1 CATEbola is a pseudonym for the clinical trial.

2 Our ethnographic study was funded by the Canadian Institutes of Health Research (PJT-148908). Ethical approval was obtained on October 2, 2018, from the hospital ethics committee at the research site.

3 Participants’ names are pseudonyms and "composite characters" combining various typical elements that enable preservation of anonymity of individual participants.
