# Correction to: Under consent: participation of people with HIV in an Ebola vaccine trial in Canada

**DOI:** 10.1186/s12910-021-00629-z

**Published:** 2021-06-01

**Authors:** Pierre-Marie David, Benjamin Mathiot, Oumy Thiongane, Janice E. Graham

**Affiliations:** 1 Faculté de Pharmacie, Université de Montréal, C.P. 6128, Succursale Centre-Ville, Montreal, H3C 3J7 Canada; 2 Département d’anthropologie, Université de Montréal, Pavillon Lionel-Groulx C. P. 6128, Succursale Centre-Ville, Montreal, QC H3C 3J7 Canada; 3 Pediatrics (Infectious Diseases), Faculty of Medicine, Dalhousie University, Halifax, B3H 4H7 Canada

## Correction to: BMC Med Ethics (2021) 22:42 10.1186/s12910-021-00606-6

It was highlighted that in the original version of this article [1] the wrong version of the additional file 1 was published (Additional file 1). The original article has been updated.

## Supplementary Information

**Additional file 1.** Interview guides.
