# Supplementary material for: Correction to: Under consent: participation of people with HIV in an Ebola vaccine trial in Canada
Source: BMC Med Ethics. 2021 Jun 1;22:69. doi: 10.1186/s12910-021-00629-z (PMC8167935; doi:10.1186/s12910-021-00629-z)
Supplement: Supplementary file 1 — Additional file 1. Interview guides. [file 12910_2021_629_MOESM1_ESM.docx]

### Global Vaccine Logics

**Canadian African Trial for Ebola Vaccine (CATEbola) Study**

### Clinical Trial Study Participants

### Interview

### We are interested in your experiences with the trial, including your thoughts and impressions of the health aspects of your involvement, as well as the social aspects. This interview has 4 parts and focuses on:

### a- Your knowledge and experience of previous clinical trials

### b- The aspects of the clinical trial that you find positive and/or difficult

### c- Your motivations to take part in the clinical trial

### d- What does Ebola mean to you?

### Can you tell me a little bit about yourself?

### How did you come to know about this trial [*Prompt: friends? colleagues? doctor? community leaders? Others? What did they tell you to bring you on board?]*

### How were you recruited? Can you tell me how it went?

### In your knowledge, what is the difference between a trial and a vaccination?

### Can you tell me about the last time you were vaccinated [*what vaccine, where, when…*]?

### Have you ever been involved in a clinical trial before? If yes, what was your experience like in that trial?

### Can you tell me about your experience of being involved in this trial? What kind of preparation did you have? What kind of support did you receive prior to starting?

### What are your motivations for being in this trial? *[Support your country? Money? Support research? Opportunity to reciprocate/give back to your doctor for treatment and care?]*

### What were your expectations for this trial prior to starting? What are your expectations now?

### What do you know about the CATEbola Study? Where does this knowledge come from? [*Friends, media, news…]* Do you feel that you know enough?

### Have you sought additional information about this trial? If so, where did you go to learn more? What kind of support do you get?

### What are the advantages and disadvantages that this trial brings to you? To your friends and family? To your local community? To national or international health?

### *[If not answered previously – see Q 7]* Did your knowledge and/or experience of the West African Ebola epidemic influence your decision to get involved in this trial? If so, please explain.

### What did you know about Ebola before you became involved in this trial?

### What have you heard about a cure for Ebola?

### Do you think people living with HIV have specific concerns with Ebola? What do you know about the risks?

### What do you hope to learn from your experience in this trial?

### Do you have any comments you would like to add about the trial, any of the questions I asked, or anything else that comes to mind?

### Clinical Trial Study Staff

### Interview

### The interview is divided into 4 parts and focuses on:

### a- Your experiences in the clinical trial (Career path, daily activities)

### b- Perceptions of the study by participants

### c- Scope of the clinical study

### d- Knowledge related to Ebola

### ___

### Can you briefly describe your career path for me? [*Training, institutional affiliation, government, tenured faculty, temporal/short term contracts?*]

### What is your role in this trial?

###

### Is this trial similar to those you have worked with before? In what ways?

### Tell me about your experience of working on this trial with respect to preparations and planning, recruitment, interactions with participants, monitoring and evaluation.

### What were your expectations for this trial prior to starting? What are your expectations now?

### What aspects of your work do you think are most important?

###

### In your opinion, what do local people think of the CATEbola trial?

###

### What kinds of communication, research and information barriers (or facilitators) have you encountered during the trial?

### What do you see as the advantages and disadvantages of this trial for the local community? For you, personally? For your friends and family? For national and international health?

### Describe what you know about the history of how this vaccine was developed. Why do you think it is important to conduct this trial here?

### What did you know about Ebola prior to your involvement in this trial? What have you learned so far? What do you still hope to learn?

### How does your knowledge of the West African Ebola epidemic affect your opinion of this trial (its relative importance, its goals, etc.)?

### What do you think will be the most important lessons that will come from this trial?
